# Signaling Pathways Involved in Diabetic Renal Fibrosis

**DOI:** 10.3389/fcell.2021.696542

**Published:** 2021-07-12

**Authors:** Yuqing Zhang, De Jin, Xiaomin Kang, Rongrong Zhou, Yuting Sun, Fengmei Lian, Xiaolin Tong

**Affiliations:** ^1^Endocrinology Department, Guang’anmen Hospital, China Academy of Chinese Medical Sciences, Beijing, China; ^2^Endocrinology Department, Guang’anmen Hospital, Beijing University of Chinese Medicine, Beijing, China; ^3^Endocrinology Department, Affiliated Hospital to Changchun University of Chinese Medicine, Changchun University of Chinese Medicine, Changchun, China

**Keywords:** signaling pathway, renal fibrosis, diabetic kidney disease, TGF-β, cross-talk

## Abstract

Diabetic kidney disease (DKD), as the most common complication of diabetes mellitus (DM), is the major cause of end-stage renal disease (ESRD). Renal interstitial fibrosis is a crucial metabolic change in the late stage of DKD, which is always considered to be complex and irreversible. In this review, we discuss the pathological mechanisms of diabetic renal fibrosis and discussed some signaling pathways that are closely related to it, such as the TGF-β, MAPK, Wnt/β-catenin, PI3K/Akt, JAK/STAT, and Notch pathways. The cross-talks among these pathways were then discussed to elucidate the complicated cascade behind the tubulointerstitial fibrosis. Finally, we summarized the new drugs with potential therapeutic effects on renal fibrosis and listed related clinical trials. The purpose of this review is to elucidate the mechanisms and related pathways of renal fibrosis in DKD and to provide novel therapeutic intervention insights for clinical research to delay the progression of renal fibrosis.

## Introduction

Diabetic kidney disease (DKD) is the most common complication of diabetes mellitus (DM), which is characterized by glomerular hyperfiltration, progressive albuminuria, and decreased glomerular filtration rate (GFR), and ultimately leads to end-stage renal disease (ESRD) (Alicic et al., 2017). According to the prevalence survey, about 30–50% of ESRD worldwide is caused by DKD (Tuttle et al., 2014). DKD has become the primary cause of ESRD in middle-aged and elderly people in China (Duan et al., 2020). The pathological changes of DKD include nodular or diffuse glomerulosclerosis, tubular inflammation, atrophy, and interstitial fibrosis (Thomas et al., 2015). Among them, renal interstitial fibrosis is a common metabolic change in the late stage of DKD, which is also a factor that promotes the progression of the disease.

**FIGURE 1:**
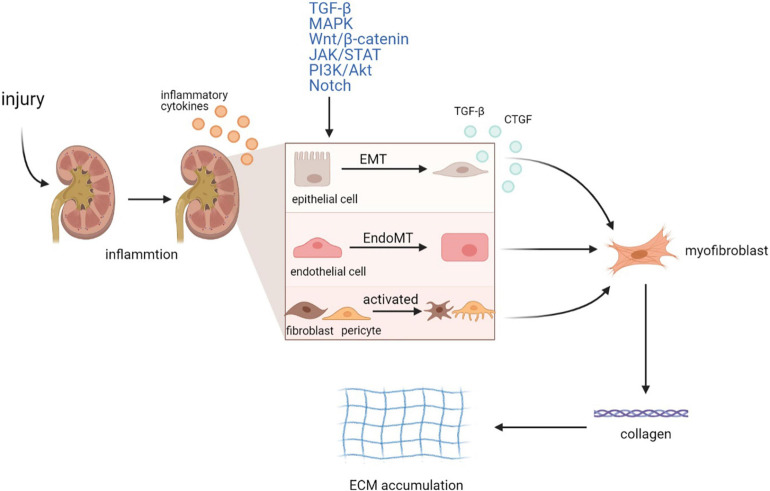
Mechanism of renal fibrosis. Injury to the kidney activates inflammatory response and promotes the secretion of inflammatory cytokines by renal cells. Then, in response to severe or persistent inflammation, renal epithelial cells and endothelial cells undergo phenotypic transitions called EMT and EndoMT, respectively, while fibroblasts and pericytes are activated. Activated intrinsic renal cells also secrete cytokines such as TGF-β and CTGF to further promote the formation of myofibroblasts. Various signaling pathways are involved in these processes, including TGF-β, MAPK, Wnt/β-catenin, PI3K/Akt, JAK/STAT, and Notch pathway. These pathological changes result in the irreversible formation of myofibroblasts, followed by the production of multiple types of collagens and the ECM accumulation, eventually leading to renal tubulointerstitial fibrosis. EMT, epithelial–mesenchymal transition; EndoMT, endothelial–mesenchymal transition.

Renal fibrosis is the deposition of fibrotic matrix and the formation of scar in response to severe or persistent injury (Humphreys, 2018). Although it is involved in the wound healing process, continued fibrosis can damage tissue structure and organ function, eventually causing renal failure (Liu, 2011). Chronic injury to the kidney promotes a variety of pathological changes, including epithelial–mesenchymal transition (EMT) (Yang and Liu, 2001; Hertig et al., 2008), endothelial–mesenchymal transition (EndoMT) (Li et al., 2009), and activation of fibroblasts and pericytes. EMT is characterized by the loss of intracellular adhesion, such as E-cadherin, and the acquisition of mesenchymal markers, such as αSMA, fibroblast-specific protein 1 (FSP1), fibronectin, collagen, and vimentin (Zhou and Liu, 2016). EndoMT is a special EMT subset that occurs in endothelial cells, which is similar to EMT (Sun et al., 2016). Fibroblasts and pericytes are always regarded as the major origin of myofibroblasts, whose activation predominantly accelerates the irreversible formation of myofibroblasts (Mack and Yanagita, 2015). These pathological processes transform renal cells into myofibroblasts, which exert their profibrotic function by secreting collagen I, III, and IV, fibronectin, and laminin, leading to extracellular matrix (ECM) accumulation and eventually resulting in tubulointerstitial fibrosis (Figure 1).

Renal fibrosis is the major outcome event of nearly all chronic kidney disease and is always considered to be irreversible and remains to be an unsolved clinical conundrum (Francois and Chatziantoniou, 2018). It is well established that many signaling pathways are involved in renal fibrosis through a variety of complex cascades. In this review, we discuss the diagnostic method and the mechanisms of renal fibrosis, especially tubulointerstitial fibrosis, and signaling pathways that are closely related to it, and summarize potential targets as well as new drugs of DKD.

## Examination and Quantification Scores of Renal Fibrosis

The native kidney biopsy is the gold standard for the diagnosis of renal interstitial fibrosis, by which the pathological process and the prognosis of patients could be evaluated more accurately (Luciano and Moeckel, 2019). According to the Banff 97 criteria (Racusen et al., 1999), an adequate cortical specimen that contains at least 10 glomeruli and 2 arteries should be obtained by kidney biopsy. Then, staining methods, such as hematoxylin and eosin (HE) stain, periodic acid-Schiff (PAS) stain, and trichrome stain, are performed to improve the identification of the degree of lesion (Farris and Colvin, 2012). Masson trichrome stain is the preferred method for diagnosis of renal interstitial fibrosis in clinical practice because of its simple operation and clear results that can be obtained under a light microscope (Street et al., 2014). Furthermore, with the introduction of immunofluorescence and electron microscopy techniques, the accuracy of qualitative diagnosis and quantitative analysis of kidney biopsy samplings has been improved (Hogan et al., 2016).

The widely used Banff 97 classification proposed by Racusen et al. (1999) divided the lesions into four grades, namely, ci0, ci1, ci2, and ci3, according to the percentage of cortical parenchyma affected. The corresponding percentage of the four levels are 0 to 5, 6 to 25, 26 to 50%, and more than 50%, respectively.

Although kidney biopsy is the only specific diagnostic method for renal fibrosis, non-invasive techniques such as diffusion tensor imaging (DTI) (Nassar et al., 2021) and molecular imaging of ECM (Bülow and Boor, 2019) are valuable in the assessment of renal interstitial fibrosis.

## Signaling Pathways Involved in Renal Fibrosis

### TGF-β Signaling Pathway

It is well accepted that the TGF-β signaling pathway plays a crucial role in fibrogenesis, especially in renal fibrosis of diabetic kidney disease. TGF-β, a key mediator of fibrosis, exerts its profibrotic effect through the activation of downstream signaling, leading to EMT, EndoMT, and myofibroblast activation, which instigates a loss of adhesion proteins and connexins under high-glucose conditions (Hills et al., 2012). Therefore, ECM accumulates on the cell surface or between cells, which is the key cause of renal fibrosis.

The three isoforms of TGF-β, TGF-β1, TGF-β2, and TGF-β3, all have fibrogenic effects on renal cells and stimulate the production of ECM proteins in renal fibroblasts, renal tubular epithelial cells, and glomerular mesangial cells (Ito et al., 2001). Among them, TGF-β1 is considered to play a major role in fibrogenesis and mediate part of the functions of TGF-β2 and TGF-β3 (Yu et al., 2003). These reports indicate a better therapeutic effect in attenuating renal fibrosis by blocking all three isoforms together. TGF-β1 binds receptor II (T beta RII) to exert its biological functions, and the disruption of T beta RII in renal fibroblasts can reduce the accumulation of ECM and inhibit fibrosis through TGF-β1-induced Smad signaling pathway (Kasuga et al., 2001; Meng et al., 2012a). CaSR, a kind of G protein-coupled receptor, could combine with TβRII to form a CaSR–TβRII complex, which is then translocated from the cell membrane to the cytoplasm. This process leads to the reduction of TβRII on the cell membrane, thereby reducing its binding to its ligand TGFβ1, inhibiting the activation of the TGF-β/Smads signaling pathway and the expression of downstream genes, and finally alleviates the ECM accumulation mediated by TGF-β1 or high glucose (Li et al., 2020a).

The TGF-β signaling pathway could be activated by high-glucose condition (Qi et al., 2007), which significantly increases the expression of TGF-β1 mRNA and induces the synthetic phenotype of mesangial cells (Liu, 2011). The injury of mesangial cells and podocytes caused by diabetic nephropathy can activate the signal transduction cascade of the TGF-β/Smad signaling pathway and stimulate the expression of TGF-1, TSP-1, and TGF-IIR in GEC, thus activating the Smad signaling pathway and leading to increased production of ECM (Kim et al., 2003). Animal studies have also demonstrated that stimulation with TGF-β1 increased the kidney expression of fibrotic genes such as collagen I, collagen IV, and fibronectin in mesangial cells and tubular epithelial cells in UUO mice (Wang et al., 2020a).

On the other hand, treatment with pyrrole-imidazole (PI) polyamides, a transcription inhibitor of TGF-β1, decreased the growth of mesangial cells, which demonstrated the role of TGF-β1 in renal fibrosis (Horikoshi et al., 2020). Treatment with anti-TGF-β antibody (αT), an antibody neutralizing the activity of all three isoforms of TGF-β, reduced the deposition of ECM and alleviated renal interstitial fibrosis (Fukasawa et al., 2004). Furthermore, the application of αT not only prevented early changes in renal histopathology and attenuated the accumulation of ECM with a short-term treatment (Sharma et al., 1996), but also reduced the expression of α1 (IV) collagen and fibronectin in later stages of diabetic nephropathy with chronic administration (Ziyadeh et al., 2000). However, a randomized, double-blind clinical study showed that using a humanized neutralizing monoclonal antibody (TGF-β1mAb) to neutralize active TGF-β1 did not slow the progression of diabetic nephropathy, and the trial was terminated early for lack of efficacy. Although TGF blockers have made some progress in animal experiments, their clinical application is still a conundrum (Voelker et al., 2017).

### The Factors That Stimulate the TGF-β Signaling Pathway

In the occurrence and development of diabetic nephropathy, many factors promote the progression of renal fibrosis by stimulating the TGF-β production, such as hyperglycemia, advanced glycation end products (AGEs), reactive oxygen species (ROS), and renin–angiotensin II–aldosterone system (RAAS).

Numerous studies have indicated that hyperglycemia is a key regulatory factor that mediates the TGF-β secretion in both *in vitro* and *in vivo* studies (Zhu et al., 2005). High glucose stimulated the expression of TGF-β1 mRNA and increased total TGF-β1 protein production in murine mesangial cells (MMCs) (Hoffman et al., 1998) and human proximal tubular cells (HPTC) (Phillips et al., 1995) in culture. Moreover, blood glucose fluctuation (BGF) plays a significant role in renal TGF-β1 gene expression; even the level of the blood glucose is in the normal range (Qi et al., 2007). Another experiment on animals demonstrated that BGF treatment markedly upregulates TGF-β1 expression, increasing the synthesis of type I collagen and inhibiting collagen degradation. Furthermore, BGF appeared to have a more harmful effect in fibrogenesis of renal cells in diabetic mice than in mice with consistent hyperglycemia (Cheng et al., 2013).

Except for the effects brought by blood glucose, AGEs also upregulated the levels of TGF-β mRNAs partly by the increasing production of ROS and subsequently induced mesangial cell hypertrophy and fibrosis (Yamagishi et al., 2003). On the contrary, the AGE cross-link breaker could reduce EMT in diabetic rats by decreasing tubular AGE and TGF-β expression (Oldfield et al., 2001). Both *in vivo* and *in vitro* studies demonstrated that AGEs induced the deposition of matrix protein *via* induction of TGF-β and Smad2/3 activation in mesangial cells, which was suppressed by the inhibitor of AGE receptor (Lee et al., 2018).

In addition, the results of the previous study suggested that the activated local RAAS contributed to high glucose-induced EMT by activating AT receptors and promoting angiotensin II production. Angiotensin II and activated AT receptors could stimulate TGF-β synthesis in the kidney and upregulated TGF-β receptors. Research also found that the AT receptor antagonist losartan partially inhibited the increases in TGF-β in rat kidney proximal tubular epithelial cell line NRK-52E (Zhou et al., 2010a), which indicated that RAAS played a pivotal role in the TGF-β signaling pathway. Currently, there is no direct way to inhibit the TGF-β system without side effects. Therefore, angiotensin-converting enzyme (ACE) inhibitors and angiotensin type 1 (AT1) receptor blockers are widely used in diabetic nephropathy at present, which partly interfere with TGF-β expression mediated by ANG II (Wolf, 2006). Although Ang II blockade alone reduces renal fibrosis, simultaneous blockade of Ang II and TGF-β takes a better effect in ameliorating renal fibrosis, suggesting that drug combinations will be a future therapeutic direction and novel measures that block the TGF signaling pathway still need further development (Yu et al., 2004).

### Downstream Targets of TGF-β Signaling

TGF-β signaling exerts its biological functions through both canonical (Smad-dependent) and non-canonical (Smad-independent) pathways (Derynck and Zhang, 2003). In the Smad-dependent pathway, the active TGF-β binds to its receptor type II and type I serine/threonine kinase receptors and thus phosphorylates Smad2 and Smad3, and oligomeric complexes are formed with Smad4, which translocates into the nucleus, regulating the transcription of target genes. Smad7, one of the inhibitory Smads (I-Smads), exerts its autoinhibitory function by inhibiting the signals from the serine/threonine kinase receptors (Miyazono et al., 2000).

**FIGURE 2:**
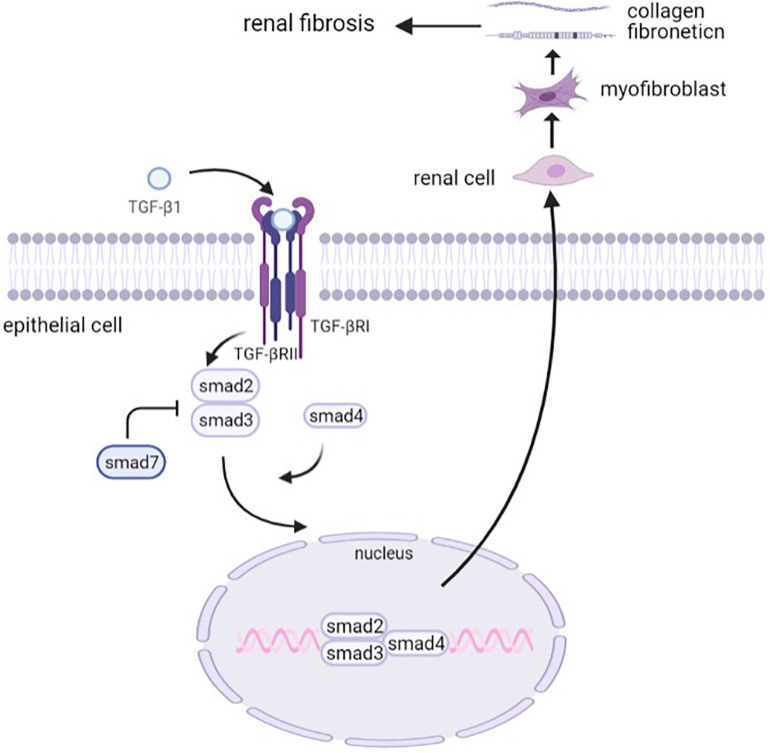
The canonical TGF-β signaling pathway. Activated TGF-β binds to its type II receptor and type I serine/threonine kinase receptors and thus phosphorylates Smad2 and Smad3. Smad7 is one of the inhibitory Smads that inhibit the signals from the serine/threonine kinase receptors. Smad2/3 then forms an oligomeric complex with Smad4, which translocates into the nucleus to regulate the transcription of target genes. The activation of the TGF-β signaling pathway drives renal cells into myofibroblasts, which secrete collagens and fibronectins to promote renal fibrosis.

In this process, Smad3 plays a major role in the development of renal fibrosis (Ju et al., 2006). It has provided evidence that the TGF-β/Smad3 signaling pathway is highly activated in renal fibrogenesis. Smad3 exhibited an obvious deposition of collagen, contributing to the development of renal fibrosis in db/db mice, whereas this process could be inhibited by Smad3 knockout (Xu et al., 2020). Further research suggested that Smad3 increased TGFβ1-induced connective tissue growth factor (CTGF) expression and decreased E-cadherin expression. It also cooperated with Smad2 to increase the expression of alpha-SMA (Phanish et al., 2006). HIPK2, a regulator of the TGF-β1/Smad3 pathway, inhibited Smad3 phosphorylation, leading to the mitigation of renal fibrosis (Liu et al., 2017), which indicated that Smad3 is a potential therapeutic target for T2DN. Although Smad2 and Smad3 are both generally considered to be crucial downstream mediators of TGF-β1, the pathological function of Smad2 and Smad3 may be different in terms of collagen matrix deposition and interstitial fibrosis. It has been demonstrated that the deletion of Smad2 had an effect in enhancing TGF-β/Smad3 signaling, which promoted collagen production and fibrosis. On the contrary, the overexpression of Smad2 attenuated this process, indicating a protective mechanism of Smad2 in TGF-mediated fibrosis (Meng et al., 2010). It corresponds to the previous results that Smad2 knockout promoted EMT progression (Ju et al., 2006; Runyan et al., 2009). Smad4, the same as Smad3, promotes fibrogenesis, which was proven by the observation of reduced collagen I expression and inhibited renal fibrosis in the Smad4 knock-out mouse model (Meng et al., 2012b). Although the downstream Smad signaling primarily promotes TGF-β-induced renal fibrosis, Smad7, an inhibitory Smad, negatively regulates fibrotic cytokines expression. The anti-fibrosis mechanism of Smad7 has been demonstrated to be a blockade of Smad2/3 activation *via* regulating the expression of microRNAs (Lan, 2008; Chung et al., 2013). In general, Smads play a crucial role in TGF-β-mediated renal fibrosis as a downstream signaling pathway (Figure 2), which provides novel and potential therapeutic targets for clinical treatment and drug discovery of renal fibrosis.

In addition to the Smad-dependent pathway, multiple non-Smad downstream signaling pathways are involved in TGF-β-mediated renal tubular fibrosis, such as the mitogen-activated protein kinase (MAPK) pathway, Wnt/β-catenin pathway, extracellular signal-regulated kinase (ERK)1/2, c-Jun N-terminal kinase (JNK), and phosphatidylinositol 3-kinase (PI3K) (Sutariya et al., 2016). These pathways cross-talk with TGF-β signaling to exert complex biological functions and together promote the progression of renal interstitial fibrosis.

### MAPK Pathway

Mitogen-activated protein kinases consist of a group of serine/threonine protein kinases, controlling various aspects of cellular functions, and can be activated in high-glucose conditions (Haneda et al., 1997). The main subgroups of MAPK are P38MAPK, ERK, and JNK, which together mediate signal transduction in fibrosis (Davis, 1994).

It is reported that the overexpression of EphA1, a modulator of fibrosis, decreased the phosphorylation of ERK1/2 and JNK in the kidney and finally alleviated renal fibrosis of diabetic nephropathy mice (Li et al., 2017c). Zhang et al. (2015) also reported that ERK1/2 MAPK signaling was involved in diabetic nephropathy to mediate fibrogenesis by regulating mesangial cell proliferation and ECM accumulation, which suggested the crucial biological function of the ERK MAPK pathway in tubulointerstitial fibrosis. Another research found that blockade of p38 MAPK exerted a beneficial effect *via* inhibiting the production of phosphorylated p38 MAPK-positive cells in the early stage of fibrosis in the treated kidney (Wada et al., 2006). Moreover, the single blockade of p38 MAPK was also demonstrated to be effective to alleviate renal fibrosis in the established fibrotic UUO model (Nishida et al., 2008). At the same time, administration of JNK inhibitor, which blocked all JNK isoforms, obviously delayed fibrosis progression *via* inhibiting accumulation of collagen IV and α-SMA + myofibroblast in an animal model (Ma et al., 2007).

Extracellular signal-regulated kinase, p38 MAPK, and JNK pathways play different but complementary roles in ECM deposition and fibrogenesis. P38 MAPK is more important in regulating Matrix metalloproteinase 1 (MMP1), while the ERK MAPK pathway mainly moderates the production of type I collagen (COL1) in high-glucose conditions (Okano et al., 2010). Moreover, P38 MAPK and JNK, but not ERK signaling, induced thrombospondin-1 (TSP-1) production, which is known to be an activator of the latent TGF-β1 complex (Naito et al., 2004). JNK markedly contributes to TGF-β1-induced CTGF mRNA expression, whereas p38 MAPK and ERK pathways take little effect (Utsugi et al., 2003).

In total, although targeting different aspects of the fibrotic process, the three subgroups of MAPK all contribute to pathological changes of renal fibrosis. Therefore, targeting the MAPK pathway as a novel and effective therapy could be a potential research direction in the future for renal fibrosis of diabetic kidney disease.

### Wnt/β-Catenin Signaling Pathway

It is well established that the Wnt signaling pathway plays multiple roles in the injury and repair of renal cells through the mediation of inflammation, fibrosis, angiogenesis, and insulin secretion (Zhou et al., 2010b; Xiao et al., 2013). The canonical pathway of Wnt is β-catenin (Harris and Peifer, 2005), through which Wnt signaling induces the expression of cyclin and glucokinase, contributing to cell proliferation and glucose sensing (Schinner et al., 2009).

Wnt signaling was activated by high glucose both in STZ-induced diabetic rats and in db/db mouse models with an upregulation of β-catenin and WNT protein level, which could be attenuated by lowering the blood glucose levels with the administration of insulin. It was found in an *in vitro* study that hyperglycemia and oxidative stress were both responsible for the activation of the Wnt pathway in renal tubular epithelial cells. The blockade of the Wnt pathway with the treatment of LDL-receptor-related protein inhibitor was demonstrated to ameliorate renal fibrosis (Zhou et al., 2012). Kameswaran et al. also observed a reduction in fibronectin and α-SMA with the treatment of inhibitor of Wnt/β-catenin signaling transduction (Surendran et al., 2005).

Further studies illuminated the detailed mechanism of Wnt/β-catenin in promoting renal fibrosis. When the signaling is “on,” the Wnt binds to the frizzled receptor, which works together with the low-density lipoprotein receptor-related proteins (LRP)5 and LRP6 co-receptors to inhibit the ubiquitination of destruction complex (Piersma et al., 2015). Therefore, the degradation activity of β-catenin by β-TrCP is limited, causing the accumulation of β-catenin (Bilic et al., 2007). Finally, β-catenin translocates from the cytoplasm to the nucleus to regulate the transcription of target genes (Kawakami et al., 2013), such as Snail and Twist (Liu, 2010), thereby promoting the EMT process. For example, Snail was involved in EMT by downregulating the expression of adhesion protein E-cadherin, which was further demonstrated by an increasing level of E-cadherin expression with the administration of Snail inhibitor (Batlle et al., 2000; Cano et al., 2000). The twist is also associated with EMT and subsequent renal fibrosis by inducing a mesenchymal phenotype in UUO mice, whereas the knockdown of Twist by short interfering RNA (siRNA) markedly attenuated EMT and fibrosis (Kida et al., 2007; Sun et al., 2009).

### PI3K/Akt Signaling Pathway

Phosphoinositide 3-kinase (PI3K) is an intracellular phosphatidylinositol kinase, also having serine/threonine (Ser/Thr) kinase activity (Fruman et al., 2017). Akt is a protein kinase, also called protein kinase B (PKB), because of its high homology with protein kinase A (PKA) and protein kinase C (PKC). PI3K is firstly activated by tyrosine kinase to transform phosphatidylinositol 4,5-biphosphate (PIP2) into phosphatidylinositol 3,4,5-triphosphate (PIP3) (Huang et al., 2018), which promotes the accumulation of Akt at the plasma membrane. Then, Thr308 of Akt is phosphorylated with the assistance of 3-phosphoinositide-dependent protein kinase 1 (PDK1). Finally, activated Akt can exert its biological function in multiple physiological processes, including cellular proliferation, differentiation, apoptosis, migration, and metabolism (Xu et al., 2015; Zeng et al., 2019).

It has been observed that PI3K/Akt signaling is associated with ECM accumulation and EMT. Therefore, it has a potential involvement in renal interstitial fibrosis (Xie et al., 2015; Shin et al., 2019). Chen et al. (2016) found that the activation of PI3K/Akt participated in renal tubulointerstitial fibrosis induced by HG condition. Furthermore, Li et al. (2017a) found that knocking down the negative regulator SHIP in human renal tubular epithelial cells (HK2 cells) led to the activation of the PI3K/Akt pathway and subsequently upregulated the expression of TGF-β1, α-SMA, and collagen type 3. Additionally, Zhu et al. also found that high-glucose condition increased the levels of phospho-Akt (Ser 473), phospho-Akt (Thr 308), CTGF, and α-SMA in both STZ-induced diabetic mice and *in vitro* human renal tubular epithelial cells. However, the upregulation of Ser 473 and Thr 308 expression was inhibited by LY294002, an inhibitor of the PI3K/Akt signal transduction pathway, followed by reduced CTGF, fibronectin, and collagen production (Zhu et al., 2016).

### JAK/STAT Signaling Pathway

The Janus kinase (JAK)/signal transducer and activator of transcription (STAT) pathway is involved in the regulation of cell proliferation, differentiation, apoptosis, and other important biological processes by activating many cytokines, growth factors, and hormones (Bhattacharjee et al., 2016). It has been established that the JAK/STAT signaling pathway is associated with renal fibrosis in both human and mouse diabetic kidney disease (Brosius, 2008). The signaling cascade of the JAK/STAT signaling pathway includes JAK activation, tyrosine phosphorylation, and STAT recruiting (Morris et al., 2018). Activation of the JAK/STAT pathway and related proteins contributed to the proliferation of renal fibroblasts and accelerated the development of renal interstitial fibrosis in rat NRK-49F cells. However, this process could be blocked by the JAK inhibitor AG490 (Sun et al., 2019). Similarly, in DKD patients, enhanced expression of JAK/STAT mRNA was observed, with the pathological changes of fibrosis. There is a possible link between JAK2, a pivotal upstream regulator of the JAK pathway, and tubulointerstitial fibrosis, which was suggested by the temporal association of upregulated JAK2 level and evolution of human DKD (Berthier et al., 2009). Moreover, a phase 2 clinical trial showed that treatment with Baricitinib, an oral selective inhibitor of JAK1 and JAK2, resulted in amelioration of albuminuria, indicating a renal protective effect of JAK signaling blockade (Tuttle et al., 2018).

A further study observed that high glucose exposure directly induced the tyrosine phosphorylation of JAK2, STAT1, STAT3, and STAT5. At the same time, the activation of JAK2 promoted the expression of downstream targets STAT1 and STAT3, but not STAT5 (Wang et al., 2002), showing the signal transduction of JAK/STAT in HG conditions. Another study showed that treatment with probiotic Lactobacillus markedly inhibited the phosphorylation of JAK2 and STAT1 in the renal cortex in STZ-induced diabetic mice and subsequently decreased the expression of α-SMA and fibronectin protein (Lu et al., 2010). In addition, the administration of selective STAT3 inhibitor reduced the profibrotic gene expression of collagen IV, TGF-β1, VEGF, and ACE in tubular epithelial cells in mouse kidneys (Zheng et al., 2019). Zhou et al. (2014) also found that the expression of JAK2, STAT3, and STAT5 markedly increased in STZ-induced mouse kidneys, whereas it decreased after treatment of suppressor of cytokine signaling (SOCS) 2, along with the attenuation of renal fibrosis formation. These results provide evidence that blockade of JAK/STAT signaling reduces collagen accumulation and profibrotic cytokine expression, resulting in the inhibition of HG-induced fibrotic response in STZ-induced mice.

Moreover, the JAK/STAT pathway has a possible involvement in AGE-induced cell proliferation and collagen production in NRK-49F cells (Huang et al., 1999; Lee et al., 2005), which account for its profibrotic function from another aspect. Huang et al. (2001) found that AGE-induced collagen expression was suppressed by either JAK2 inhibitor or STAT1 and STAT3 decoy oligodeoxynucleotides (ODNs) in NRK-49F cells and thus concluded that JAK2-STAT1/STAT3 was responsible for the induction of collagen and promoted renal fibrogenesis.

### Notch Signaling Pathway

Notch signaling consists of four transmembrane receptors (Notch1–Notch4), two Jagged family ligands (JAG1 and JAG2), and three delta-like ligands (DLL1, DLL3, and DLL4) in the mammalian system (Hu et al., 2015). It can be reactivated in pathological conditions and involved in a variety of processes, including cellular proliferation, apoptosis, and EMT (Kopan and Ilagan, 2009). Upon the combination of the Notch signaling pathway ligand and the receptor, Notch transforms to the activated form Notch intracellular domain (NICD), which enters the nucleus to regulate the expression of downstream targets and trigger ECM and EMT, and ultimately results in renal fibrogenesis in diabetic kidney disease (Bonegio and Susztak, 2012).

The activation of the Notch signaling pathway was observed both in tubular interstitial fibrosis (TIF) patients and in TIF mouse models. Moreover, the Notch pathway was proved to be both necessary and sufficient for the occurrence and development of TIF. Tian et al. (2018) demonstrated reno-protective functions of Gliquidone, which took effect by blocking the Notch/Snail signaling pathway to delay renal fibrosis. Additionally, with the administration of γ-secretase inhibitor, a pharmacological inhibitor of Notch activation, the cascade reaction of the Notch signaling pathway was blocked (Nishad et al., 2019). The blockade of Notch resulted in the amelioration of renal fibrosis in the folic acid-induced (FA-induced) TIF mouse model, as reflected by the decreased level of fibronectin, collagen, and vimentin (Bielesz et al., 2010). Jing et al. (2020) also found that administration of DAPT, an inhibitor of the Notch pathway, reversed HG-induced protein expression of Jagged1, PGC-1α, and Drpl in renal tubular epithelial cells. It indicated that Notch signaling pathway might accelerate renal fibrosis by regulating oxidative damage and mitochondrial dysfunction.

Among the downstream genes of the Notch signaling pathway, the Snail plays the most important role in inducing fibrosis. It acts as a bridge for inducing EMT in tubular epithelial cells and activating the Notch signaling pathway (Grande et al., 2015). The expression of the Snail promoter is stimulated by the Notch pathway in a dose-dependent manner (Sahlgren et al., 2008), and its overproduction directly leads to the decreased expression of E-cadherin and increased production of alpha-SMA, MMP-2, and MMP-9 (Saad et al., 2010). Yang et al. (2017) found that suppressing the Notch/Snail axis activation with the treatment of berberine (BBR) increased E-cadherin as well as decreased α-SMA level, protecting renal tubular cells against EMT and renal fibrosis both *in vivo* and *in vitro*.

### Cross-Talks Among Signaling Pathways

In the occurrence and development of renal fibrosis, these pathways above not only play an independent promoting role but also have complex interactions with each other. The complicated cross-talks among them further illuminate the mechanism of renal tubulointerstitial fibrosis in diabetic kidney disease. Cross-talks involving the TGF-β signaling pathway have been highlighted because the TGF-β pathway plays a predominant role in the fibrogenic process and interacts with nearly all fibrosis-related pathways.

### Cross-Talk Between MAPK and TGF-β Pathways

In terms of the cross-talk between TGF-β and MAPK, Beek et al. (Chin et al., 2001) found that treatment with exogenous TGF-β1 in mesangial cells markedly induced phosphorylation of p38 MAPK and ERK1/ERK2. The activation of the former one could be blocked by an inhibitor of the TGF-β type I receptor. Blocking of the latter one markedly inhibited the phosphorylation of ERK1/2 induced by high glucose and at the same time attenuated the level of TGF-β1 expression and resulted in alleviation of EndMT (Yu et al., 2017). Utsugi et al. (2003) also found that levels of phosphorylated threonine and tyrosine of JNK and p38MAPK increased by adding TGF-β1 in human lung fibroblasts. Therefore, these findings demonstrated that TGF-β1 has stimulating effects on all the main subgroups of MAPK. Further study found that the ERK/p38 MAPK pathway activity played a critical role in maximal induction of Smad activity by TGF-β1 (Li et al., 2004). The blockade of ERK inhibits R-Smad-dependent transcriptional activation, leading to a reduction in TGF-β-induced transcriptional activity (Hayashida et al., 2003). Also, there is a positive feedback loop between JNK and TGF-β/Smad (Grynberg et al., 2017). The activation of the TGF-β/Smad pathway stimulates JNK in the epithelial cell, while JNK directly induces the phosphorylation of the linker of Smad3, thereby promoting the transcriptional activity of Smad (van der Velden et al., 2011). In addition, extracellular MAPK activation induced by factors such as TNF-α, angiotensin II, and IL-1 can stimulate latent TGF-β in mesangial cells *via* TSP-1 (Naito et al., 2004) or activator protein 1 (AP-1) (Lee et al., 2006; Rui et al., 2012). In summary, there is a positive cross-talk between MAPK and TGF-β/Smad signaling pathways, which causes synergistic enhancement of TGF-dependent responses.

### Cross-Talk Between Wnt and TGF-β Pathways

TGF-β/Smad and Wnt/β-catenin pathways were found to promote renal fibrosis concertedly or independently. The TGF-β pathway could activate the canonical Wnt pathway mainly by downregulating the level of Wnt antagonist Dickkopf-1 (DKK1) (Akhmetshina et al., 2012). The decrease of DKK1 upregulated Wnt1-dependent β-catenin expression (Wang et al., 2011). In addition to the Wnt1-dependent pathway, TGF-β repressed WT1, a negative regulator of β-catenin, to promote the secretion of β-catenin and its target genes, such as MMP-9, Snail1, PAI-1, and Fsp1 (Chang et al., 2008; Kim et al., 2010). TGF-β also promotes β-catenin dephosphorylation and activation by inactivating glycogen synthase kinase-3β (GSK-3β) (Zeng et al., 2005; Dai et al., 2011). Furthermore, the cross-talk between these pathways can be partly indicated by the binding of β-catenin with Smad3 in renal tubular epithelial cells, which results in the progression of EMT, suggesting the profibrotic effects that β-catenin brings for TGF-β1 (Tian et al., 2013). In addition, Wnt signaling pathway components, especially Wnt11, are regulated by a Smad3-dependent mechanism, thus promoting the activation of mesenchymal marker genes, such as Snail1, Zeb1, Pai1, and SMA. It finally leads to pathological changes, including fibroblast proliferation and ECM deposition (Zhang et al., 2012).

The fact that pharmacological blockade of either TGF-β or Wnt would be sufficient to inhibit the other one further demonstrated the synergistic effect of TGF-β and Wnt/β-catenin. Stellor et al. found that deleting TβRII in proximal tubule epithelial cells inhibited the expression of TGF-β and, at the same time, blocked the β-catenin signaling (Nlandu-Khodo et al., 2017). In return, Hoi et al. (2020) identified that the WNT/β-catenin inhibitor, ICG-001, as well as its derivatives blocked TGF-β-induced phosphorylation of Smad2/3, thus ameliorating renal fibrosis triggered both by TGF-β and by Wnt pathways.

### Cross-Talk Between PI3K/Akt and TGF-β Pathways

Accumulating evidence has revealed that the PI3K/Akt pathway has a close relationship with TGF-β signaling in promoting ECM deposition and renal fibrosis. The PI3K/Akt pathway was found to be activated in renal tubular cells exposed to high-glucose condition, followed by increased expression of TGF-β1 and production of ECM (Hao et al., 2011). Inhibition of the PI3K/Akt pathway by the chemical inhibitor LY294002 or a small hairpin RNA vector downregulated TGF-β1 production and indirectly alleviated the accumulation of ECM and collagen (Wu et al., 2007). Chen et al. also found that with the treatment of carboxyl-terminal modulator protein (CTMP), an endogenous inhibitor of Akt (Chae et al., 2005), the expression of TGF-β and α-SMA was markedly reduced in cultured renal tubular cells (Chen et al., 2016). On the other hand, PI3K/Akt serves as a pivotal downstream regulator of TGF-β1, whose activation markedly promotes TGF-β1-induced EMT and collagen production (Bakin et al., 2000; Runyan et al., 2004). Kattla et al. (2008) also found that administration of TGF-β1 led to activation of PI3K and Akt, which could be demonstrated by increased phosphorylation levels of Ser473 and GSK-3β. In terms of the mechanism by which TGF-β activates PI3K/Akt, researchers have different opinions. Some researchers hold the view that TGF-β directly activates PI3K and subsequently stimulates Akt production (Higaki and Shimokado, 1999), while others consider TGF-β-induced activation of PI3K as p38MAPK dependent (Horowitz et al., 2004).

### Cross-Talk Between JAK/STAT and TGF-β Pathways

In addition to the well-known Smad family, TGF-β also activates JAK1, STAT1, STAT3, and STAT5 to regulate the occurrence of fibrosis (Dong et al., 2016). In return, STAT3 can enhance fibrosis partly by stimulating TGF-β expression (Ogata et al., 2006). Researchers observed that the TGF-β-induced phosphorylation of STAT3 was dependent on the binding of JAK to TβRI in the early stage and was mediated by Smad3 and TβRI kinase activity in the late phase (Itoh et al., 2018; Wiegertjes et al., 2019). It was verified that blockade of JAK2 by either JAK2 antisense or specific inhibitor AG-490 inhibited TGF-β protein expression and fibronectin production in glomerular mesangial cells. Also, depletion of STAT1 by corresponding antisense, but not STAT3, reduced HG-induced TGF-β expression (Wang et al., 2002), suggesting that JAK2-STAT1 signaling has a great influence on the TGF-β pathway.

### Cross-Talk Between Notch and TGF-β Pathways

Studies have shown that the Notch pathway is mediated by the TGF-β pathway in renal fibrosis, and these two signaling pathways play a mutual promoting role both *in vivo* and *in vitro*. On the one hand, the TGF-β pathway was reported to upregulate the ligand of Notch, such as jagged1 (Liu et al., 2013) and Hey1 (Zavadil et al., 2004) in kidney cells. Moreover, Wang et al. (2017) found that the TGF-β inhibitor reduced the expression of Notch and its target genes, including Notch1, Hes1, and Hes5, in the rats with tissue fibrosis, demonstrating a stimulating function of the TGF-β pathway in Notch signal transduction. On the other hand, a previous study reported that treatment with (−)-epigallocatechin gallate (EGCG) inhibited the Notch pathway in kidney cells, which is mainly through the inhibition of TGFβRII and Smad3 (Zhu et al., 2020). Xiao et al. (2014) also found that the administration of the Notch inhibitor dibenzazepine (DBZ) inhibited the activation of the Notch pathway, as well as the expression of TGF-β and the phosphorylation of Smad2 and Smad3, which markedly ameliorated the severity of renal fibrosis. It was also observed that the inhibitory effect of sh-Notch1 or GSI on the Notch pathway increased the expression and activity of MMP-2 and MMP-9, reduced the level of TGF-β1, and inhibited the expression of type IV collagen and laminin in mouse podocytes (Yao et al., 2015).

Furthermore, studies observed a direct link between TGF-β and Notch signaling pathways in renal fibrosis. NICD, an active form of Notch that is closely related to the extent of tubulointerstitial fibrosis (Murea et al., 2010), was found to interact directly with Smad3, which was enhanced by TGF-β administration (Klüppel and Wrana, 2005). In addition, Blokzijl et al. (2003) discovered that Hes-1, a confirmed Notch target gene, also acts as a direct downstream gene of the TGF-β pathway. The activation of Hes-1 by either the TGF-β or Notch pathway contributes to the fibrogenesis in kidney cells (Zavadil et al., 2004). In summary, these all indicate that Notch signaling and TGF-β signaling have a functional synergism in regulating renal fibrosis.

### Cross-Talk Between Notch and Wnt Pathways

It has been reported that the Wnt pathway has a positive cross-talk with the Notch pathway (Bertrand et al., 2012; Perkins et al., 2016). Estrach et al. (2006) concluded that JAG1, a known ligand of the Notch pathway, also acts as a β-catenin target gene that is stimulated by the activation of Wnt/β-catenin signaling. To further verify it, Chen et al. (2010) inhibited the Wnt signaling and observed a reduction in JAG1 expression. Mödder et al. (2011) also found that Wnt10b could promote the activation of Wnt and Notch signaling, indicating that Wnt/β-catenin signaling performed as an upstream mediator of the Notch pathway.

**FIGURE 3:**
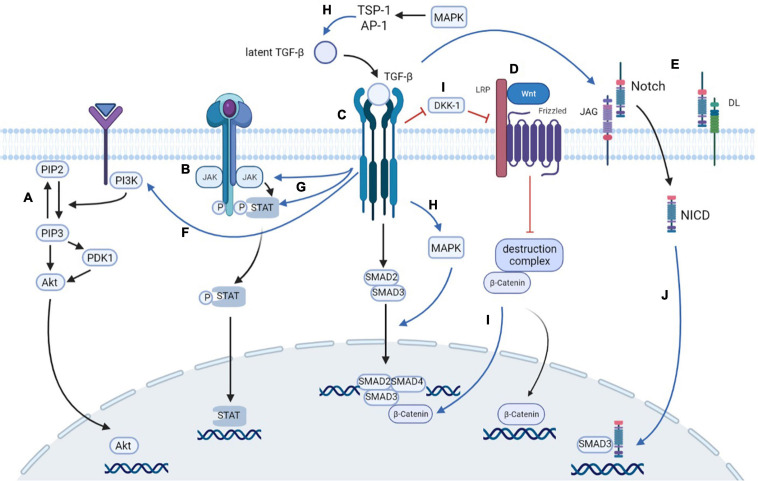
Cross-talks among signaling pathways. **(A)** PI3K/Akt signaling pathway: PI3K is activated to transform PIP2 into PIP3, which promotes the accumulation of Akt with the assistance of PDK1 at the plasma membrane. Activated Akt then translocates into nucleus to exert its biological function. **(B)** JAK/STAT signaling pathway: The signaling cascade of JAK/STAT signaling pathway including JAK activation, tyrosine phosphorylation, and STAT recruiting. Then, the STAT is transported to nucleus to regulate gene transcription. **(C)** TGF-β/Smad signaling pathway: Activated TGF-β binds to its receptors and thus phosphorylates Smad2 and Smad3. Smad2/3 then forms an oligomeric complex with Smad4, which translocates into the nucleus to regulate the transcription of target genes. **(D)** Wnt/β-catenin signaling pathway: The Wnt binds to the frizzled receptor, which works together with the LRP5 and LRP6 co-receptors to inhibit the ubiquitination of destruction complex. Therefore, the degradation activity of β-catenin is limited, causing the accumulation of β-catenin, which translocates from the cytoplasm to the nucleus to regulate the transcription. **(E)** Notch signaling pathway: Upon the combination of the receptor Notch, and the ligand JAG and DL, Notch transforms to the activated form NICD, which enters the nucleus to regulate the expression of downstream targets. **(F)** TGF-β directly activates PI3K and subsequently stimulates Akt production. **(G)** TGF-β can activate both JAK and STAT, and the activation of JAK subsequently stimulates the phosphorylation of STAT3. **(H)** The activation of the TGF-β/Smad pathway stimulates MAPK, while MAPK directly induces the phosphorylation of the linker of Smad3, thereby promoting the transcriptional activity of Smad. Extracellular MAPK activation can stimulate latent TGF-β in mesangial cells *via* TSP-1 or AP-1. **(I)** TGF-β pathway activates Wnt pathway by downregulating the level of Wnt antagonist DKK1. β-catenin can bind with Smad3, promoting the transcription of Smads. **(J)** TGF-β pathway upregulates the ligand of Notch, such as JAG, to transform Notch to NICD. NICD can interact directly with Smad3, which is enhanced by TGF-β administration. PI3K, phosphoinositide 3-kinase; PIP2, phosphatidylinositol 4,5-biphosphate; PIP3, phosphatidylinositol 3,4,5-triphosphate; PDK1, 3-phosphoinositide-dependent protein kinase 1; JAK, Janus kinase; STAT, signal transducer and activator of transcription; LRP, low-density lipoprotein receptor-related proteins; NICD, notch intracellular domain; TSP-1, thrombospondin-1; AP-1, activator protein 1; DKK-1, Dickkopf-1.

In conclusion, there is a close connection between the TGF-β pathway and other pro-fibrosis pathways, and the mechanism of fibrosis progression with TGF-β as the core has been established (Figure 3).

## New Therapies for Renal Fibrosis

### New Drugs Designed for Renal Fibrosis

Roxadustat, a new drug designed for renal anemia (Chen et al., 2019b), has been shown to inhibit hypoxia-inducible factor (HIF) and alleviate the progression of renal fibrosis (Packer, 2020). Li et al. (2020b) have implemented animal research to figure out that Roxadustat may retard the progression of fibrosis by regulating Akt/GSK-3β-dependent Nrf2 activation. Li et al. designed a randomized, multicenter, and active-controlled study (NCT02652806) of the efficacy of Roxadustat in anemia treatment in CKD patients (Chen et al., 2019a), but there is still a lack of clinical evidence to support this antifibrotic effect of Roxadustat.

Paricalcitol (PC) is a vitamin D analog that retains similar vitamin D bioactivity but has fewer side effects (Sprague et al., 2003; Fryer et al., 2007). Tan et al. (2009) found that Paricalcitol inhibited the expression of fibronectin, collagen, vimentin, as well as Snail1 in UUO mice, showing a remarkable amelioration in fibrogenesis. Nolan et al. (2015) observed that PC affected the progression of renal fibrosis *via* targeting the TGF-β1/Smad2 pathway, Notch/Jagged1 pathway, and PI3K pathway in cultured human renal epithelial cells. In comparison, Chung et al. (2017) figured out that PC might also alleviate renal fibrosis through the MAPK signaling pathway. In addition, PC shows a synergistic effect on the blocking of RAAS by ACEI (Martínez-Arias et al., 2021), which possibly participates in the mitigation of EMT and renal fibrosis. A randomized clinical trial (PALIFE NCT01820078) has been designed to explore the effect of Paricalcitol on fibrosis on chronic renal diseases, but the trial has been terminated.

It has been well established that overactivation of the mineralocorticoid receptor (MR) results in renal fibrosis and is closely linked to the progression of renal injury in DKD (Shrestha et al., 2019; Patel et al., 2020). Therefore, new drugs targeting MR are considered as potential therapies that slow the progression of renal fibrosis. Finerenone is a novel, non-steroidal, selective MR antagonist (MRA) with fewer negative effects on serum potassium and renal function in comparison with steroidal MRAs (Rico-Mesa et al., 2020). This medication is in phase 3 clinical trials and shows promising results in CKD progression, composite kidney outcome, and cardiovascular events (Agarwal et al., 2020). The ARTS-DN trial (NCT01874431) conducted by Kabris et al. proved that DKD patients with the administration of Finerenone for 90 days had lower urine albumin creatinine ratio (UACR) in a dose-dependent way compared to placebo (Bakris et al., 2015). The FIGARO-DKD (NCT02545049) trial designed by Agarwal et al. is a randomized, double-blind, placebo-controlled, parallel-group, and multicenter phase 3 study. It assessed the efficacy and safety of Finerenone on the reduction of all-cause mortality and the onset of kidney failure and its influence on eGFR and UACR in DKD patients (Ruilope et al., 2019). The study has been completed, but the results have not been published yet. Another randomized, double-blind phase 3 study (FIDELIO-DKD NCT02540993) has similar inclusion criteria and intervention with FIGARO-DKD. The results indicated that the experimental group had lower composite kidney outcomes (kidney failure, a sustained ≥ 40% decrease in eGFR from baseline, or renal death) (Filippatos et al., 2021) and reduced risks of CKD progression and cardiovascular events compared to placebo (Bakris et al., 2020).

Pirfenidone is an antifibrotic drug widely used in the clinical treatment of idiopathic pulmonary fibrosis (IPF) (Nathan et al., 2019). However, recent studies have figured out that Pirfenidone also exerts its anti-fibrotic effect in renal fibrosis. Takakura et al. (2012) observed that Pirfenidone ameliorated renal interstitial fibrosis partly through decreasing the expression of TGF-β-induced collagen, fibronectin, CTGF, and plasminogen activator inhibitor type 1 (PAI-1) in rat proximal tubular epithelial cells. In addition to the TGF-β pathway, Li et al. (2017c) found that Pirfenidone could attenuate EMT and renal fibrosis by antagonizing the MAPK pathway both *in vivo* and *in vitro*. Therefore, Pirfenidone can inhibit related signaling pathways and reduce the expression of fibrotic markers to slow the process of renal fibrosis (Chen et al., 2013). A randomized phase 3 study has been designed to evaluate the efficacy of Pirfenidone on GFR and albuminuria in DKD patients^1^. This study will assess the improvement of GFR and the changes of 24-h urine microalbuminuria and TGF-β concentrations to determine the therapeutic effects Pirfenidone takes in the progression of DKD and renal fibrosis. It is still on the recruiting stage and has no result yet. Another phase 2 clinical study also in recruitment is to assess the change from baseline in renal fibrosis by diffusion-weighted magnetic resonance imaging (DW-MRI) and urinary markers in CKD patients (^2^ NCT04258397). Moreover, the trial NCT00063583 has already provided evidence that subjects assigned to the Pirfenidone group (1200 mg/day) had higher mean eGFR than placebo after 6 months of treatment (Sharma et al., 2011; Table 1).

**TABLE 1:** New drugs that target renal fibrosis.

Number	Randomized controlled trial (NCT)	Drug	Actual enrollment	Status	Locations
1	NCT02652806	FG-4592	305 patients	completed	China
2	PALIFE (NCTO1820078)	Paricalcitol	127 patients	terminated	Spain
3	ARTS-DN (NCTO 1874431)	Finerenone	823 patients	completed	United States
4	FIGARO-DKD (NCT02545049)	Finerenone	7437 patients	completed	United States
5	FIDELIO-DKD (NCT02540993)	Finerenone	5734 patients	completed	United States
6	NCT02689778	Pirfenidone	62 patients	recruiting	Mexico
7	TOP-CKD (NCT04258397)	Pirfenidone	200 patients	recruiting	United States
8	NCT00063583	Pirfenidone	77 patients	completed	United States

**Number**	**Primary outcomes**		**Results**		

1	Hb mean change from baseline		Roxadustat led to a numerically greater mean (±SD) change in hemoglobin level from baseline to weeks 23 through 27 (0.7 ± 1.1 g/dl) than epoetin alfa (0.5 ± 1.0 g/dl)		
2	Albuminuria in proteinuric CKD patients		No result		
3	Change of urinary albumin-to-creatinine ratio from baseline to 90 days		UACR reduction: Finerenone: for 7.5 mg/day, 0.79 [90% CI, 0.68–0.91; *p* = 0.004]; for 10 mg/day, 0.76 [90% CI, 0.65–0.88; *p* = 0.001]; for 15 mg/day, 0.67 [90% CI, 0.58–0.77; *p* < 0.001]; for 20 mg/day, 0.62 [90% CI, 0.54–0.72; *p* < 0.001]		
4	Time to first occurrence of the following composite endpoints: onset of kidney failure, a sustained decrease in estimated glomerular filtration rate (eGFR) of ≥40% from baseline over at least 4 weeks and renal death Time to all-cause mortality Change in UCAR from baseline to month 4		No result		
5	Time to the first occurrence of the composite endpoint of onset of kidney failure, a sustained decrease of estimated glomerular filtration rate (eGFR) ≥40% from baseline over at least 4 weeks and renal death		The composite kidney outcome (kidney failure, a sustained ≥40% decrease in eGFR from baseline, or renal death) reduced in the Finerenone group		
6	Effect of oral Pirfenidone (1800 mg) in albuminuria and glomerular filtration rate		No result		
7	Change from baseline in kidney fibrosis, as assessed by diffusion-weighted magnetic resonance imaging (DW-MRI) and urinary markers of tubulo-interstitial fibrosis		No result		
8	The change in renal function from baseline to the end of the study period (12 months)		The mean eGFR increased in the Pirfenidone 1200-mg/day group (+ 3.3 ± 8.5 ml/min per 1.73 m^2^) whereas the mean eGFR decreased in the placebo group (−2.2 ± 4.8 ml/min per 1.73 m^2^; *p* = 0.026 versus Pirfenidone at 1200 mg/day)		

*Data from clinical trials (https://clinicaltrials.gov/).*

Moreover, these drugs have shown anti-fibrosis benefits in other organs besides the kidney. Roxadustat was found to take effect in the pulmonary fibrosis mice model *via* the inhibition of the TGF-β/Smad signaling pathway (Huang et al., 2020). Similarly, Paricalcitol showed relevant beneficial effects in the alleviation of liver fibrosis (Reiter et al., 2019), peritoneal fibrosis (Ko et al., 2019), and myocardial fibrosis (Panizo et al., 2013) by blocking the TGF-β pathway. Pirfenidone is a broad-spectrum anti-fibrosis drug that has been approved for the treatment of IPF (Rogliani et al., 2016). It inhibits the myofibroblast differentiation and collagen production in the lung by blocking the overexpressed pathways, including the TGF-β signaling pathway (Li et al., 2018), PI3K-Akt pathway (Kurita et al., 2017), and Wnt/β-catenin pathway (Ballester et al., 2019). It is also involved in liver fibrosis by inhibition of the TGF-β pathway and downregulation of Smads (García et al., 2002).

In addition to their anti-fibrosis effects, it is also important to understand the adverse effects of these drugs. According to the previous clinical trials, Roxadustat was generally well tolerated with few treatment-emergent adverse events (TEAEs). Common TEAEs were diarrhea, vomiting, contusion (Akizawa et al., 2020a), back pain, and nasopharyngitis (Akizawa et al., 2020b). It was also reported that patients taking Roxadustat had relatively higher risks of upper respiratory infection, hyperkalemia (Chen et al., 2019a), and metabolic acidosis (Chen et al., 2019b). With the administration of Paricalcitol, patients are more likely to develop hypercalcemia, hyperphosphatemia, and excessive suppression of parathyroid hormone, which could return to normal with the reduction of drug dose (Oblak et al., 2018). The most reported adverse events of Finerenone were diarrhea, muscle spasms, blood creatine phosphokinase increased, dizziness (Bakris et al., 2015), nasopharyngitis, constipation, and bronchitis (Katayama et al., 2017). In terms of adverse effects of Pirfenidone, gastrointestinal disorder (King et al., 2014), skin-related disease, and fatigue (Maher et al., 2020) were most reported. However, the overall percentage of patients with these adverse events was only slightly higher than in the placebo group, indicating an acceptable level of drug safety.

### RNA Interference (RNAi) Therapy in Renal Fibrosis

RNA interference technique is an emerging therapeutic by which messenger RNA (mRNA) could be degraded selectively and the expression of specific proteins was inhibited (Saw and Song, 2020). Hence, RNAi is involved in renal fibrosis by inhibiting the overexpression of related genes, most commonly TGF-β1 (Takabatake et al., 2005) along with its downstream genes, such as CTGF (Ren et al., 2015) and proliferator-activated receptor coactivator-1 alpha (PGC-1α) (Wang et al., 2018) and phosphorylation of Drp1 at serine 616 (p-Drp1S616) (Wang et al., 2020b). Researchers found that small interfering RNA (siRNA)-mediated gene silencing effectively suppressed the expression of corresponding mRNA and protein and thus significantly inhibited the secretion of fibrogenic factors and delay the progression of renal fibrosis (Khaja et al., 2016; Isaka, 2018). The RNAi strategy offers a potential therapy for renal fibrosis, but the research is still in its initial stage and requires more attention.

These studies suggest that some progress has been made in the treatment of organ fibrosis, especially renal fibrosis. The process of renal fibrosis is divided into three stages, namely, inflammatory reaction, fibrosis formation, and scar formation (Liu, 2011). Blocking the transformation of renal intrinsic cells into myofibroblasts and degrading fibrous tissue by targeting the fibrotic pathway can block or even reverse the progression of the disease in the first two phases (Sun et al., 2016). However, once renal fibrous tissue develops into scar tissue, it can never be reversed (Nastase et al., 2018). Therefore, we emphasized that renal fibrosis may be blocked and renal function may be improved with effective anti-fibrosis therapy in the early stage of fibrosis.

Kidney biopsy is difficult to perform in clinical trials due to its complexity, high cost, and considerable pain to patients. Therefore, there is a lack of solid evidence to support the anti-fibrosis effects of these drugs. Future studies with repeated biopsies should be conducted to demonstrate whether tubulointerstitial fibrosis can be reversed with novel therapies and to reveal the specific mechanisms of them.

## Conclusion

Renal fibrosis is a pivotal pathological change in DKD and markedly increases the mortality rate of late-stage DKD patients. In this paper, we discussed various signaling pathways involved in renal fibrosis, including the TGF-β, MAPK, Wnt/β-catenin, PI3K/Akt, JAK/STAT, and Notch pathways. These pathways all play a significant role in the accumulation of ECM, the expression of collagen and fibronectin, and the secretion of other related proteins. Moreover, an increasing number of novel therapies are under clinical study, and many efforts have been made to delay or even try to reverse the progression of renal fibrosis. Despite this progress, the exact effects of new drugs on renal tubulointerstitial fibrosis are still not clear and need to be further studied employing renal biopsies. With the development of modern medical technology, renal fibrosis is expected to become a reversible process, and the prognosis of DKD is expected to be improved.

## Author Contributions

YZ and DJ contributed to the conception and design of the study. YZ wrote the first draft of the manuscript. DJ, XK, RZ, and YS wrote the sections of the manuscript. FL and XT guided the writing of this article. All authors contributed to manuscript revision, read, and approved the submitted version.

## Conflict of Interest

The authors declare that the research was conducted in the absence of any commercial or financial relationships that could be construed as a potential conflict of interest.

## Footnotes

1 https://clinicaltrials.gov/NCT02689778

2 https://clinicaltrials.gov/TOP-CKD
